# Construct validation of a Frailty Index, an HIV Index and a Protective Index from a clinical HIV database

**DOI:** 10.1371/journal.pone.0201394

**Published:** 2018-10-17

**Authors:** Iacopo Franconi, Olga Theou, Lindsay Wallace, Andrea Malagoli, Cristina Mussini, Kenneth Rockwood, Giovanni Guaraldi

**Affiliations:** 1 Infectious Diseases Unit, The University of Modena and Reggio Emilia, Modena, Italy; 2 Geriatric Medicine Research Unit, Department of Medicine, Dalhousie University& Nova Scotia Health Authority, Halifax, Nova Scotia, Canada; University of Brescia, ITALY

## Abstract

**Background:**

Standard care for HIV clinical practice has started focusing on age-related problems, but despite this recent change physicians involved in HIV care do not often screen HIV patients for frailty. Our aim was to construct three indexes from an HIV clinical database (i.e. Frailty Index, (FI), HIV Index, (HIVI), and Protective Index (PI)) and to assess levels of frailty, HIV severity and demographic and protective lifestyle factors among HIV patients.

**Methods and findings:**

We included data from 1612 patients who attended an Italian HIV clinic between September 2016 and December2017 (mean±SD age: 53.1±8 years, 73.9% men).We used 92 routine variables collected by physicians and other health care professionals to construct three indexes: a 72-item FI (biometric, psychiatric, blood test, daily life activities, geriatric syndromes and nutrition data), a 10-item HIVI (immunological, viral and therapeutics) and a 10-item PI (income, education, social engagement, and lifestyle habits data)(the lower the FI and HIVI scores, and the higher the PI scores, the lower the risk for participants).The FI, HIVI and PI scores were 0.19±0.08, 0.48±0.17 and 0.62±0.13, respectively. Men had higher FI (0.19±0.08 vs 0.18±0.08; *p* = 0.010) and lower HIVI (0.47±0.18 vs 0.50±0.15; *p* = 0.038) scores than women. FI and HIVI scores both increased 1.9% per year of age (*p* < 0.001), whereas the PI decreased 0.2% per year (*p*<0.050). In addition, the FI score increased 1.6% and the PI score decreased 0.5% per year of HIV infection (*p* < 0.001).

**Conclusion:**

It is feasible to assess levels of frailty, HIV severity and protective lifestyle factors in HIV patients using data from a clinical database. Frailty levels are high among HIV patients and even higher among older patients and those with a long duration of HIV. Future studies need to examine the ability of the three indices to predict adverse health outcomes such as hospitalization and mortality.

## Introduction

The number of people older than 50 years living with HIV has increased from 76.000 in 1990 to 880.000 in 2016 in Western and Central Europe and North America [1]. This increase likely reflects both higher rates of new diagnoses in people older than 50 years (7.4% in 1990 vs 15.6% in 2017) [2] and longer life expectancy due to better antiretroviral therapies [3]. An important consequence is that more people who live with HIV are susceptible to common geriatric syndromes which represents a challenge to conventional medical management. For example, Green and colleagues [4] found that nearly half of people living with HIV were aged 50 or older, of whom 30–50% were affected by geriatric syndromes such as delirium, incontinence and falls. This suggests that many people aging with HIV will be frail. Possible explanations of this clinical phenomenon include the association between HIV specific factors, chronic inflammation and dysregulation in the immune system along with a higher burden of multiple concomitant noninfectious age-related comorbidities (e.g. cardiovascular disease, chronic kidney disease, chronic obstructive pulmonary disease, diabetes mellitus and osteoporosis). These HANA (HIV-Associated-Non-AIDS) chronic conditions occur at a younger age in HIV positive individuals compared with their HIV-counterparts and are associated with higher rates of polypharmacy [5, 6], long-term ART toxicity, and changes in body fat and muscle composition. They are also related to higher prevalence of behavioral and social risk factors and worse socio-economic conditions in HIV positive patients [7–11]. Indeed, as reported by Althoff and colleagues [12], HIV infected adults are at higher risk of myocardial infraction, end stage renal disease and non-AIDS related cancer. To this point it is important to mention that prevalence of risk behaviors, such as smoking, drinking and having an hypercaloric diet, and comorbid conditions known to be well established cardiovascular risk factors such as hypertension and dyslipidemia, is higher among people living with HIV [7–11, 13, 14]. Finally, the authors of another cohort study (the AGEhIV Cohort Study) described higher prevalence of age-associated comorbidities and respective risk factors when comparing HIV infected with uninfected individuals [15].

Frail people experience a poor resolution of homeostasis even after minor stress events. Frailty affects multiple organ systems, in particular, it has been associated with the development of dementia and cognitive impairment [16, 17], lower levels of testosterone [18] and higher levels of diurnal cortisol [19], sarcopenia [20], and immune system dysfunction that makes the frail individual susceptible to recurrent infections [21]. Therefore, frailty can be understood as a state of increased vulnerability among people of the same age which increases risk of falls, disability, dependency, hospitalization, institutionalization, and death [16]. Many clinical tools have been suggested to operationalize frailty. Among them, the Frailty Phenotype and Frailty Index are the most applied in HIV medicine. Frailty Phenotype consists of five different items such as: self-reported weight loss and exhaustion, impairments at standard physical performance tests (walking gait speed, chair to stand test) and reduced physical activity (evaluated through questionnaires) [22]. Another well-validated assessment tool in geriatric medicine is the Frailty Index (FI). This tool has been applied in various clinical settings and is based on the accumulation of health deficits approach [23]. Multiple health deficits or problems can be included in the FI such as signs and symptoms of age-related diseases, impairments in activities of daily living, and comorbidities. Our group has previously studied correlations and associations between these two health measures and clinical outcomes in PLWH with similar results, however, FI showed stronger association with age, nadir CD4 count, comorbidities, falls, and disability [24]. Having a single score that is able to depict global health status and trajectory with data collected in everyday practice and stored in clinical databases might add useful information to clinical evaluation, beyond HIV related problems. A patient’s FI score is calculated by the ratio of health deficits present, dividing by all variables evaluated, and it ranges from 0 to 1 [25]. The FI has been shown to predict adverse health outcomes better than chronological age [26]. FIs developed in clinical settings other than HIV medicine have been able to predict adverse outcomes such as mortality, morbidity, falls and re-admission [27–29]. In the HIV field, our group has previously developed a 37-item FI based mostly on blood test variables which was able to predict multimorbidity and mortality in HIV positive people independent of markers of HIV disease severity [30]. Its use allows quantification of the risk of adverse outcomes in people living with HIV. Accordingly, VACS Index, which is a deficit accumulation index like the FI but was not developed to measure frailty, has been utilized as a prognostic tool for survival [31]. In addition, another study has used an FI in an HIV population to demonstrate association between frailty and higher levels of innate immune activation [32].

In contemporary HIV care, initial goals of therapy such as undetectable HIV viral load and the achievement of normal CD4+ T-cell count represent only the starting point of a general health assessment [33]. Great concern has risen among HIV researchers and physicians due to the lack of consensus on defining “geriatric age” in PLWH [4, 34, 35]. This is due to the higher prevalence of geriatric syndromes and frailty at a younger age in PLWH. Therefore, it is important to assess healthy aging in PLWH and to screen for frailty among these subjects. Growing consensus suggests that implementing geriatric tools in clinical practice to depict health trajectories across aging HIV cohorts may be useful [35]. This has led to the development of a new discipline within HIV medicine termed “geriatric HIV medicine” [36]. As part of an ongoing effort within our medical center to establish and promote geriatric HIV medicine, we are collecting a wide range of clinical information across multiple domains such as functional ability, cognition, physical and mental health, and socio-economic conditions. We also decided to include FP items as different variables of this new FI, in order to finally merge clinical information [37]. Indeed, exploring these domains represent the main innovation in this new FI compared to the previous one which was, as mentioned above, more laboratory oriented, and did not include many geriatric health variables.

The aim of our study was to construct three indices (i.e. Frailty Index, (FI), HIV Index, (HIVI), and Protective Index (PI)) using data stored in our clinical database in order to assess levels of frailty, HIV severity and demographic and protective lifestyle factors, which might positively affect the life of individuals aging with HIV. We also assessed the construct validity of these measures by evaluating their relationship with age and duration of HIV and by comparing the FI with other FIs validated in other community dwelling samples other than HIV. Our future goal is to assess the predictive validity of these three indexes with the clinical endpoints of hospital admission, institutionalization, disability and death.

## Methods

### Design and study sample

The Modena HIV Metabolic Clinic is a multidisciplinary tertiary level care center which was established in 2004 at The University of Modena and Reggio Emilia in Italy. Our clinic serves patients with HIV from this region as well as across Italy. People from other regions are followed by a physician in their hometown and receive clinical care annually. The Modena HIV Metabolic Clinic uses a comprehensive assessment technique to examine and follow the metabolic profiles, comorbidities and, more recently, the physical, emotional, cognitive and social function of people living with HIV [38, 39]. Data are collected from blood tests, nuclear medicine DXA scan, self-reported surveys, face-to-face interviews and clinical evaluation by physicians, occupational therapists, dieticians and psychologists.

In our study, we included data from 1612 consecutively recruited individuals who visited the clinic from September 2016 to December2017. The study has been approved by the Research Ethics Board of The University of Modena and Reggio Emilia and all participants provided written informed consent.

### Frailty index (FI)

An FI was built according to standard procedures described by Searle and colleagues [40]. Candidate deficits had to explore multiple health domains and organ systems such as blood tests, emotional status, ability to handle daily activities, physical performances, nutrition and comorbidities. In addition, to be included in the FI, variables had to meet the following criteria: (i) associated with age (not just attributes related to age, such as “greying hair”); (ii) increased prevalence with age; (iii) < 20% missing cases; (iii) when combined, measures had to capture multiple organ dysfunction; (iiiii) ordinarily collected and stored in our clinical database at every visit at MHMC. A total of 72 items across different domains were selected to construct the FI, which included: urinary incontinence [41], hearing loss, falls and consequent fractures, bio-morphological data including systolic blood pressure, diastolic blood pressure, Body Mass Index, waist circumference and sarcopenia [42], global nutrition, protein intake, central obesity, bone mineral density, self-reported unintentional weight loss, polypharmacy [43], gait speed, chair stand test [44], 17 blood tests including hormone levels, 10 mental health questions measured with the CES-D depression scale [45], 8 instrumental activities of daily living items [46, 47], 6 global health status questions from the EQ5D5L scale [48], 4 respiratory function questions from the St. George Respiratory questionnaire [49],and 8 comorbidities [50–54] (Table A in S1 Table).

Every variable was recoded into a 0 (no deficit) to 1 (presence/full deficit) score. Participants with mild or moderate impairment received a corresponding score that indicated the proportion of the deficit (e.g. 0.25). Variables with less than 1% or more than 80% of the participants having the health deficit were excluded. For example, a single IADL question concerning the ability to handle medication had to be excluded because the deficit was present in less than 1% of the study population [40]. The FI score was calculated as the sum of all deficits scores divided by the sum of all variables evaluated for each participant. Scores ranged from 0 (absence of all deficits) to 1 (presence of all deficits). An FI was not calculated for participants who had missing data for more than 20% of variables (14 missing variables; 105 participants, 6.5% of study population) [55]. The FI was used in the analysis as both continuous and dichotomous variable using the (≥)0.25 cut-point. This cut-off point was chosen because of the nature of community dwelling samples [56]. FI scores in these populations tend to be lower than clinical samples and are associated with age. This reflects the concept that levels of frailty in community dwelling samples are lower than clinical ones [56].

### HIV Index (HIVI)

An HIV Index (HIVI) was constructed using data collected as part of standard HIV clinical practice according to current clinical national and international guidelines [57, 58]. Variables included were: Centers for Disease Control and Prevention (CDC) 1993 revised classification system for HIV infection [59], CD4+ Nadir, duration of HIV, time between diagnosis and ARV start, 3^rd^ line of ARV or more (as a surrogate of drug resistance or toxicity), HIV/AIDS related cancers, presence or absence of lipodystrophy (using MACS criteria) [60], current CD4+ T-cell count, HIV Viral load, and CD4-CD8 ratio. Each variable was recoded into a 0 (no deficit) to 1(full expression of a deficit) (See Table B in S1 Table for HIVI variables coding). Similar to the construction of the FI, the HIVI was calculated as the ratio between the number of HIV-related health deficits present divided by the total number of HIV variables collected (i.e. *n* = 10 HIV variables). HIVI was not calculated when participants had more than 20% of missing data (*n* = 50 participants, 3.1% of study population). We also constructed a shorter HIVI excluding duration of HIV so that we can investigate the relationship between HIVI and duration of HIV. Since this is the first study where we attempted to create an HIV index, no cut-off points have been established to differentiate grades of HIV disease severity.

### Protective Index (PI)

To construct a PI we used the demographic and lifestyle variables routinely collected in The Modena HIV Metabolic Clinic. In agreement with previous work from our research group [61] we considered demographic and lifestyle choices as “protective factors”. Variables were chosen from those included in common Social Vulnerability Indices [62, 63]. The following variables were included in the PI: ethnicity, level of education, profession, income, physical activity [64], injection drug use (past or current), marital status, domestic partnership, alcohol use (high consumption: more than two glasses of wine/beer/spirits for five or more days per week; moderate consumption: more than two glasses of wine/beer/spirits for two to five days a week; mild: more than two glasses of wine/beer/spirits once a week or less), smoking habit (in order to clearly identify individuals with no deficit, (never smokers) and to separate them from the rest of the study population, we decided to lower the limit of “heavy smokers” from 20 pack-years to 10, therefore our coding for smoking habit is as follows: high consumption: more than 10 pack-years; moderate consumption: less than 10 pack- year; no consumption: never smokers). Ethnicity was recoded as “Italian vs non-Italian”. This is because language barriers can impede normal social functioning and can also influence access to health care and communication with health care professionals. In addition, ethnicities other than Italian-Caucasian included in the MHMC study population typically includes people from low-income countries, who marginalized and live in poor socioeconomic environments. Each variable was recoded into a 0 (full deficit) to 1(no deficit—protective) (See Table C in S1 Table for coding of variables). The PI was not calculated when participants had more than 20% of missing data (*n* = 426 participants, 26.4% of study population). As with the HIVI, this is the first study to propose a PI in an HIV population, no cut-points have been established to differentiate levels of ‘protection’.

### Statistical analysis

**Fig 1:**
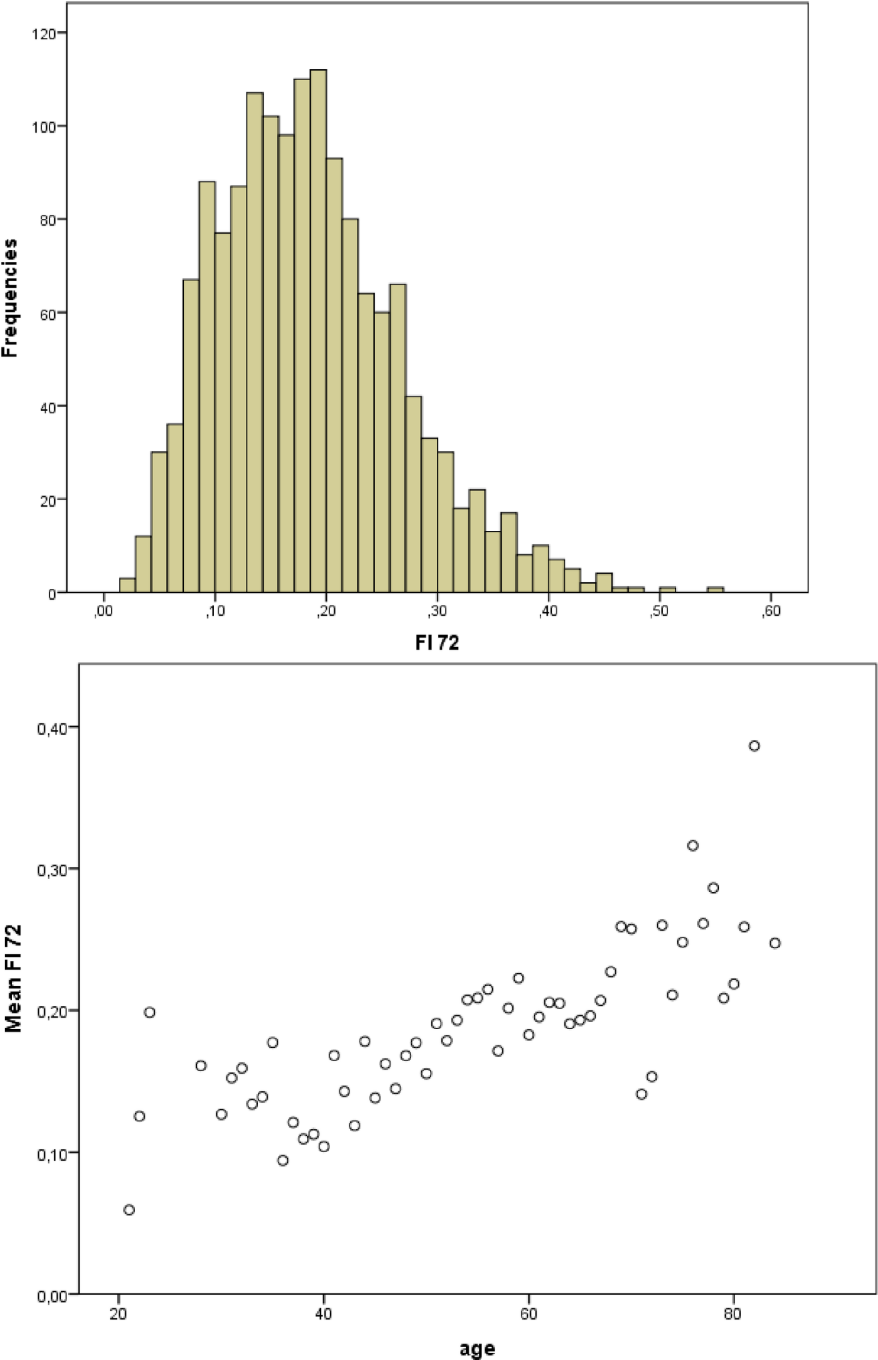
FI characteristics. (A) Distribution of FI; (B) Association between FI and age.

**Fig 2:**
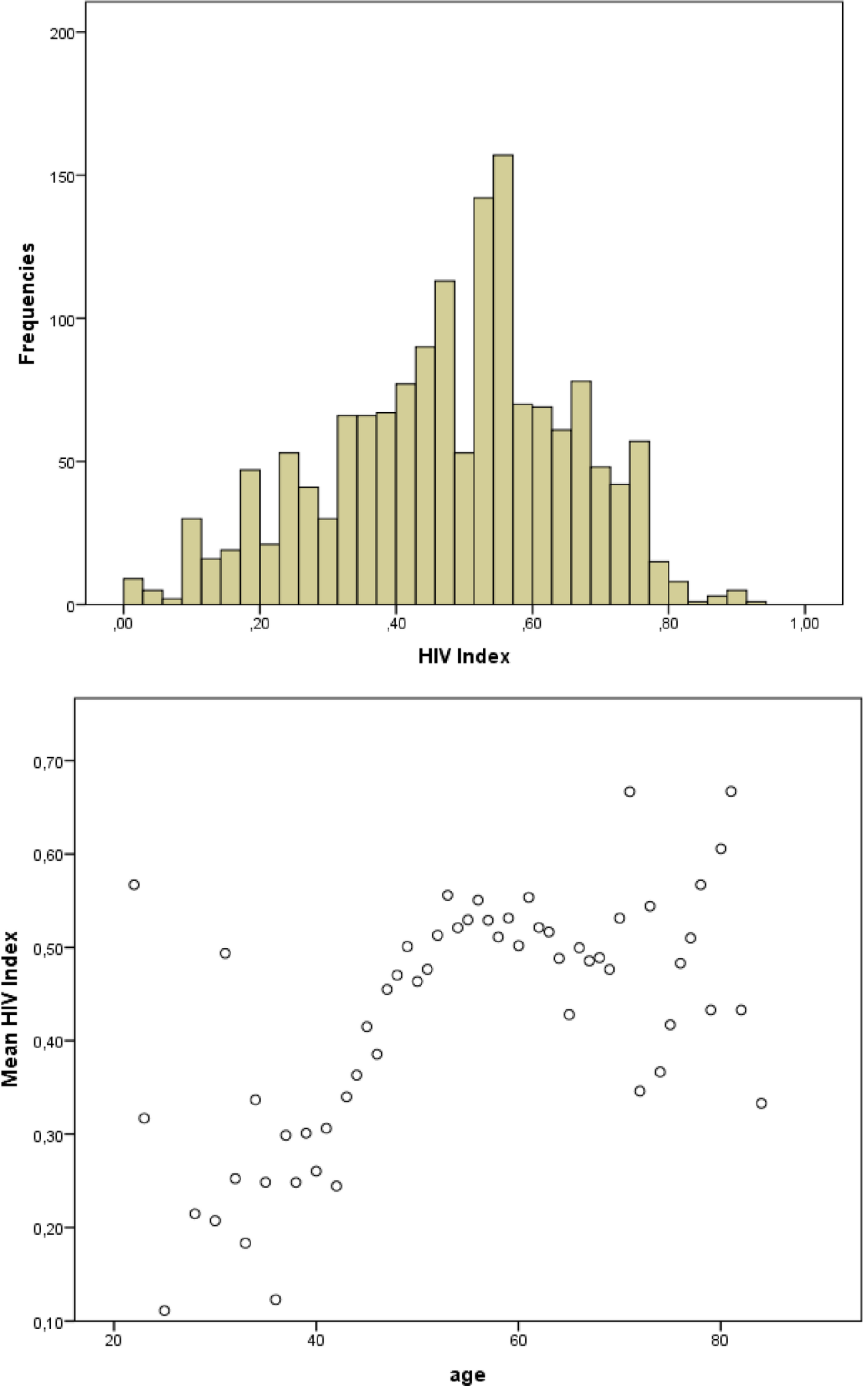
HIVI characteristics. (A) Distribution of HIVI; (B) Association between FI and age.

**Fig 3:**
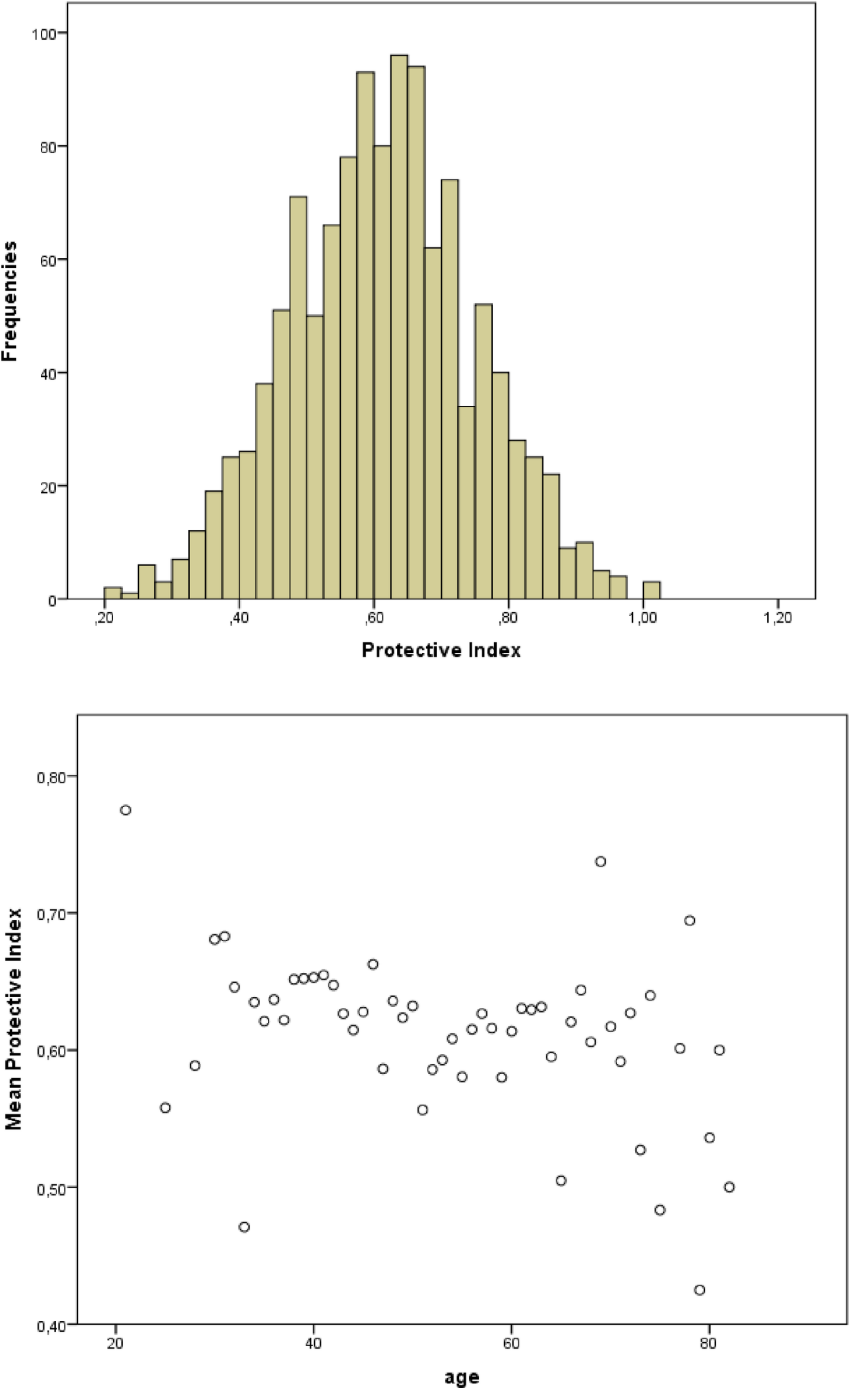
PI characteristics. (A) Distribution of PI; (B) Association between FI and age.

We assessed the distribution of the three indices using histograms (Figs 1A, 2A and 3A). Patients were divided into groups according to age (less than ≤40, 41–50, 51–60 and>>60 years of age) and duration of HIV infection (≤10, 11–20,21–30, >30 years of infection). We chose 0.25 as the FI cut-off point to determine frail individuals because 0.25 is the most commonly used FI cut point in the literature [65]. Prevalence of frailty and mean FI, HIVI, and PI scores were calculated for the study population in relation to sex, age groups and duration of HIV. Differences in mean index scores were examined using one-way ANOVA with adjustment for multiple pair-wise comparisons. Differences in prevalence of frailty within age groups, duration of HIV groups and sex were examined using non-parametric tests. Univariate and multiple linear regression analyses were performed to investigate the relationship between the index score with age (FI, HIVI, PI) and duration of HIV (FI, PI). SPSS version 24 (IBM Corp, Armonk, NY, USA) was used to perform statistical analyses.

## Results

Our study included 1612 people, the mean age was 53.1±8.0 years (median 53, range21-84 years) and most were men (79.3%, *n* = 1157). The median duration of HIV was 22.0 (0–32) years, respectively. We were able to construct the FI for 1507 (93.5%) participants since the patients with more than 20% missing variables were excluded. The lowest FI value was 0.02 and the highest was 0.55. Using the 0.25 FI cut-point we estimated that the prevalence of frailty among the participants in our study was 20.2%. Both mean FI and prevalence of frailty increased with age and with longer duration of HIV (*p <* 0.001) (Table 1 and Fig 1B). Men had higher mean FI scores than did women (0.19±0.08 vs 0.18±0.08, *p <* 0.05). Univariate linear regression analysis between the natural log of FI (lnFI) and age showed a 1.9% increase in lnFI score per year of life. Univariate linear regression analysis between the natural log of FI (lnFI) and duration of HIV infection showed a 1.6% increase in lnFI score per year of infection (*p*< 0.001).

**Table 1:** FI score and prevalence of frailty (FI≥0.25) according to sex, age and duration of HIV.

	Mean ±SDFI	*N* (%) of FI >0.25	Mean ±SDHIVI	Mean ±SDPI
All patients (*N* = 1612)	0.19±0.08	325 (20.2%)	0.48±0.17	0.61±0.14
**Sex**				
Men (*N* = 1191)	0.19±0.08	238 (21.3%)	0.47±0.18	0.61±0.13
Women(*N* = 421)	0.18±0.08^a^	68 (17.5%)	0.50±0.15^a^	0.61±0.14
**Age groups**				
≤40 years (*N* = 97)	0.12±0.06	1 (1.1%)	0.27±0.15	0.64±0.12
41–50 years (*N* = 422)	0.16±0.08^a^	49 (12.5%)^a^	0.42±0.18^a^	0.63±0.12
51–60 years (*N* = 829)	0.20±0.08^a^^,^^b^	183 (23.6%)^a^^,^^b^	0.52±0.15^a^^,^^b^	0.59±0.13^b^
>60 years (*N* = 264)	0.21±0.07^a^^,^^b^	73 (29.4%)^a^^,^^b^	0.50±0.17^a^^,^^b^	0.61±0.14
**Duration of HIV**				
≤10 years (*N* = 215)	0.15±0.08	18 (8.9%)	0.28±0.14^+^	0.64±0.12
11-20years (*N* = 366)	0.17±0.08^a^	45 (13.1%)	0.43±0.18^+^^,^^a^	0.62±0.12
21-30years (*N* = 690)	0.19±0.08^a^^,^^b^	149 (23%)^a^^,^^b^	0.51±0.15^+^^,^^a^^,^^b^	0.62±0.13
>30years (*N* = 294)	0.22±0.08^a^^,^^b^^,^^c^	85 (30.4%)^a^^,^^b^	0.50±0.15^+^^,^^a^^,^^b^^,^^c^	0.56±0.14^a^^,^^b^^,^^c^

^a^statistical significant different from first category

^b^statistical significant different from second category

^c^statistical significant different from third category

^+^shorter HIVI excluding duration of HIV

A total of 1562 (96.7%) subjects met the criteria for the construction of HIVI. Mean±SD HIVI was 0.48±0.17, median HIVI 0.48, the lowest value was 0.00 and the highest value was 0.92. Mean HIVI was higher in women than in men (0.50±0.15 vs 0.47±0.18, *p <* 0.05) and HIVI increased with age (Fig 2B). Univariate linear regression analysis between the natural log of HIVI and age showed an increase of 1.9% in lnHIVI per year (*p* < 0.001). Using the shorter HIVI, which excluded duration of HIV from the index, we found that the lnHIVI increased1.7% per year of infection (*p* < 0.001).

A total of 1186 (73.6%) subjects met the criteria for the construction of PI. Mean±SD PI was 0.61±0.14). Median PI was 0.61, the lowest PI was 0.20 and the highest was 1.00. No statistically significant difference in PI score between men and women was found (0.61±0.13 vs 0.61±0.14 *p >* 0.05). The PI showed overall reduction with increasing age (0.2% of decrease in lnPI per year of age, *p* = 0.019) (Fig 3B) and statistically significant differences in PI were found between participants of 41–50 years of age and those 51–60 years (PI = 0.63±0.12 vs 0.59±0.13, *p* <0.001). Differences in PI were also statistically significant when comparing those with the longest duration of HIV (more than 30 years of infection) with the other 3 groups (*p <*0.05). Univariate linear regression between natural log of PI and duration of HIV showed an average decrease of 0.5% in the PI score per year of infection (*p* < 0.001).

## Discussion

We showed that it is feasible to assess levels of frailty, HIV severity and protective factors in HIV patients using data collected from a clinical database. Levels of frailty and poor lifestyle factors are high among HIV patients and even higher among older HIV positive patients and those with a long duration of HIV. Since HIV negative and positive populations are different in terms of comorbidities, geriatric syndromes and social and environmental factors [4, 18], no age limit has been set yet to define the elder population among HIV positive individuals, therefore, we decided to include all patients regardless of age. Due to the short period of observation, major clinical endpoints could not be used to validate these three health indexes, however, construct validation was performed, assessing the relationship with age and the rates of health deficit accumulation in comparison with other FI’s validated with clinical endpoints as hospital admission, institutionalization, disability and death [25, 27–29]. Considering our population is a community dwelling sample, levels of frailty were lower than those obtained from clinical samples as expected [25], as with the association with age (0.7 increase in mean FI per year), even if rates of deficit accumulation were slightly lower (1.9% of deficit accumulation per year vs 2.9 in other longitudinal observational studies).

We have analyzed and collected data from multiple health domains involving different health care professionals and obtaining 92 variables overall from 1612 patients. Not all included variables are routinely collected across clinics, but most of them are easy to assess, at least once per year. We were able to successfully build a 72-item FI, a 10-item HIV-Index, and a 10-item Protective index, and analyze their correlations with sex, age and duration of HIV. Compared to the previous 37-item FI validated in our clinic, this new FI explores more health domains, including IADLs, geriatric syndromes, comorbidities, depression quotient, and malnutrition, which are important comprehensive assessments. Distribution of the FI was right-skewed, the prevalence of frailty increased with chronological age and longer duration of HIV, and FI scores were higher in men than in women. We also created two new indices, one, the HIVI, using HIV-specific variables from the sample population, and the other, the PI, using demographic and lifestyle variables that could have a protective effect on individuals’ overall health. The HIVI frequency displayed a right skewed distribution. Women had higher rates of HIVI scores than men, and mean HIVI score increased across all four age groups. Duration of HIV was originally included as an item in the HIVI but a shorter version of HIVI without duration years of infection was calculated and found to be associated with duration of HIV. On the contrary, PI was left-skewed and showed a statistically significant reduction when comparing people aged 41–50 years with people 51–60 years. The non-significant difference in PI for the other two age groups could be related to the smaller sample size. Interestingly, rates of protective factors accumulation reduced by 0.2 and 0.3 per year of age and HIV infection, respectively. No sex differences were found in PI.

### Strengths and limitations

Our results should be interpreted with caution. Due to the short period of study we were not able to investigate the association between the FI, HIVI, and PI with common clinical outcomes such as hospitalization and mortality. Further, variables included in our study were routinely collected as part of a comprehensive health assessment in our HIV clinic, but may not be feasible to collect in general practices. The complexity of the data and variables analyzed, and the broad age range of the study population would make it difficult to find a HIV negative control group for comparison, therefore the lack of a control group is another study limitation. Our data showed trends similar to what has been reported in population studies; FI scores increased with age, had a limit below 0.7, and a mean increase of 1.9% of deficit accumulation per year, which is slightly less than the average rates reported in community based longitudinal observational studies (2.9%) [56]. This can be explained by the relatively younger median age of the participants (53 years old, range 21–84) in our study compared to other study populations [15, 27, 66], which were analyzed to operationalize the FI. The sample population used to construct the PI was smaller than the sample populations of both the FI and HIVI, because socio-environmental data was collected a few months after the beginning of the study period.

Despite these limitations, our medical center continues to evaluate a high number of health variables exploring biological, infectious, socio-environmental domains in a high-quality manner and is the first to create three different indices to assess overall health in the HIV aging population. Each of the FI, HIVI and PI show correlation with age (FI and HIVI had a positive correlation with age whether PI had a negative correlation), and even more importantly with duration of HIV infection, suggesting that duration of HIV infection might be a good predictor of biological aging in HIV individuals. These health indices could be interpreted as measures of the general health and biological age of an individual. The large clinical sample, the high number of variables included, the relatively few missing data, and the multidisciplinary approach behind the concept of the three indices leads us to conclude that the FI, HIVI and PI might represent important measures, which could be considered an integral part of a comprehensive health assessment in the HIV aging population.

### Conclusions and clinical implications

Our study demonstrates that it is feasible to build health indexes from data stored in a clinical database. With the available data we were able to create a 72-item FI exploring multiple health domains, a 10 item HIV index and a 10 item Protective Index. Despite the great number of variables analyzed, the vast majority of them, especially the HIV and socio-economic variables, are easy to assess and can be obtained through questionnaires. Despite no cut-off points validated for HIVI and PI, these two indexes are based on health variables, which have important clinical implications on patients’ health. Once built, health indexes are able to provide an overall score for the health of the patient and can also be used to track health changes over time. Therefore, clinical utility of these health indexes lies in their ability to combine multiple variables across different health domains to create a single score.

Future studies are needed to evaluate causal relationships between HIVI, PI and FI and how the HIVI and PI can predict the onset and progression of frailty. A key priority moving forward will be to validate and operationalize these three indices with clinical outcomes in external clinical settings. We aim to investigate this in depth in order to create a feasible clinical assessment tool, which is able to rapidly and easily assess general health status in the HIV aging population.

## Supporting information

S1 Table Health variables. (A) Frailty Index variables. (B) HIV Index variables. (C) Protective Index variables.
(DOCX)
