# Supplementary material for: Construct validation of a Frailty Index, an HIV Index and a Protective Index from a clinical HIV database
Source: PLoS One. 2018 Oct 17;13(10):e0201394. doi: 10.1371/journal.pone.0201394 (PMC6192552; doi:10.1371/journal.pone.0201394)
Supplement: S1 Table — (A) Frailty Index variables. (B) HIV Index variables. (C) Protective Index variables. (DOCX) [file pone.0201394.s001.docx]

|  | | |
| --- | --- | --- |
| **Deficit** | **Coding** | **Frequencies**  **N (%)** |
| Urinary Distress Inventory (UDI6) | 0: no distress  0.5: mild-moderate distress  1: severe distress/disability | 0=1224(85.2)  0.5=77(5.4)  1=136(9.5)  Missing=175(10.9) |
| Impact of urinary incontinence on QoL (IIQ) | 0: no incontinence  0.5: mild-moderate incontinence  1: severe incontinence | 0=1272(88.25)  0.5=85(5.9)  1=80(5.6)  Missing=175(10.9) |
| Hearing loss questionnaire | 0: no handicap  0.5: mild-moderate handicap  1: severe impairment | 0=1157(80.5)  0.5=227(15.8)  1=53(3.7)  Missing=175(10.9) |
| Did you ever fall in the previous year? | 0: no reported fall in the previous year  1: reported fall in the previous year | 0=1259(87.1)  1=186(12.9)  Missing=167(10.4) |
| Have you experienced at least one bone fracture after a fall in the previous year? | 0: No fall resulting in fracture in the previous year  1: at least one reported Fall resulting in fracture in the previous year | 0=1361(85.4)  1=232(14.6)  Missing=19(1.2) |
| Diastolic blood pressure | 0: Diastolic pressure 60-90mmHg  1: Diastolic blood < 60mmHg or > 90 mmHg | 0=1361(85.4)  1=232(14.6)  Missing=19(1.2) |
| Systolic blood pressure | 0: Systolic blood pressure 90-140 mmHg 1: Systolic blood <90mmHg or >140mmHg | 0=1364(85.6)  1= 229(14.4)  Missing=19(1.2) |
| Waist circumference | 0: normal waist circumference Women <=88cm, Men <=102cm  1: abnormal waist circumference Women >88cm, Men >102 cm | 0=1200(74.4)  1=395(24.8)  Missing=17(1.1) |
| BMI | 0: BMI =18.5-25.0 kg/m^2^  1: BMI <18.5kg/m^2^ or >25.0kg/m^2^ | 0=913(57.5)  1=676(42.5)  Missing=23(1.4) |
| Unintentional weight loss of more than 4.5 Kg in the previous year | 0: No unintentional weight loss of more than 4.5 Kg reported in the previous year  1: Unintentional weight loss of more than 4.5 Kg reported in the previous year | 0=1575(98.2)  1=29(1.8)  Missing=8(0.5) |
| Walking gait speed during 4-meters test | 0: above lowest quartile of performance on a timed 4-meter walk test  1: below lowest quartile of performance on a timed 4-meter walk test | 0=1464(95.9)  1=63(4.1)  Missing=85(5.3) |
| Chair Stand test (5 times) | 0: above lowest quartile of performance  1: below lowest quartile of performance | 0=1294(80.3)  1=234(14.5)  Missing=84(5.2) |
| Polypharmacy | 0: Less than 5 drugs  0.5: From 5 to 7 drugs  1: more than 7 drugs | 0=1075(74.4)  0.5=252(17.5)  1=117(8.1)  Missing=168(10.4) |
| Central Obesity | 0: DXA trunk fat/[armfat+legfat] <1.96  1: DXA trunk fat/[armfat+legfat] >1.96 | 0=1333(84.5)  1=245(15.5)  Missing=34(2.1) |
| Protein intake | 0: Protein Intake 0.9-1.5g/kg/daily  1: Abnormal Protein Intake < 0.9g/kg/daily or > 1.5g/kg/daily | 0=824(51.1)  1=596(37)  Missing=192(11.9) |
| Lumbar | 0: Normal bone  0.5: Osteopenia  1: Osteoporosis | 0=732(45.8)  0.5=698(43.7)  1=167(10.5)  Missing=15(0.9) |
| Femur | 0: Normal bone  0.5: Osteopenia  1: Osteoporosis | 0=726(45.6)  0.5=802(50.4)  1=63(4)  Missing=21(1.3) |
| Sarcopenia – height adjusted appendicular skeletal muscle index (ASMi) | 0: >5.45 for Women and >7.26 for Men  1: <5.45 for Women and <7.26 for Men | 0=767(49.4)  1=787(50.6)  Missing=58(3.6) |
| White Blood Cells count | 0: 4.000-10.900/mL  1: < 4.000/mL or > 10.900/mL | 0=1451(91.2)  1=140(8.8)  Missing=21(1.3) |
| Hemoglobin | 0: 12-16g/dL  1: <12g/dL or >16g/dL | 0=1341(84.3)  1=250(15.7)  Missing=21(1.3) |
| Platelets | 0: 150000-450000/mL  1: abnormal platelets levels <150.000/mL or >450.000/mL | 0=1355(85.2)  1=236(14.8)  Missing=21(1.3) |
| Glycemia | 0: Glycemia 70-90mg/dL  1: Glycemia >90mg/dL or <70mg/dL | 0=1174(73.6)  1=421(26.4)  Missing=17(1.1) |
| Triglycerides | 0: Normal triglycerides <150mg/dL,  1: High triglycerides >150 mg/dL | 0=1050(65.8)  1=545(34.2)  Missing=17(1.1) |
| Total Cholesterol levels | 0: Total Cholesterol levels <200mg/dL  1: Total Cholesterol levels > 200mg/dL | 0=1046(65.5)  1=551(34.5)  Missing=15(0.9) |
| HDL | 0: HDL levels >43mg/dL  1: HDL levels <43mg/dL | 0=1077(67.7)  1=514(32.3)  Missing=21(1.3) |
| LDL | 0: LDL levels <115mg/dL  1: LDL levels > 115mg/dL | 0=857(54)  1=731(46)  Missing=24(1.5) |
| AST/GOT | 0: GOT levels 1-31U/mL  1: GOT levels > 31U/ml | 0=1309(82.2)  1=284(17.6)  Missing=19(1.2) |
| ALT/GPT | 0: GPT level <31 U/ml  1: GPT levels >31 U/mL | 0=1171(73.4)  1=425(26.4)  Missing=16(1) |
| GGT | 0: GGT levels < 38 U/mL  1: GGT levels > 38 U/mL | 0=1124(69.7)  1=463(29.2)  Missing=25(1.6) |
| Bilirubin | 0: bilirubin 0.16-1.10mg/dL  1: bilirubin <0.16mg/dL or >1.10mg/dL | 0=1411(88.7)  1=179(11.3)  Missing=22(1.4) |
| Blood Phosphorus | 0: Blood Phosphorus => 2.5 and <=5.1mg/dL  1: Blood Phosphorus <2.5mg/dL or >5.1 mg/dL | 0=1330(87.2)  1=195(12.1)  Missing=87(5.4) |
| Creatinine | 0: creatinine levels >=0.5 to <=1.2 mg/dL  1: creatinine levels < 0.5 or >1.2 mg/dL | 0=1407(88.2)  1=189(11.8)  Missing=16(1) |
| Parathyroid hormone | 0: parathyroid hormone >=15 to <=88pg/mL  1: parathyroid hormone<15pg/mL or >88 pg/mL | 0=1445(89.6)  1=109(6.8)  Missing=58(3.6) |
| Thyroid stimulating hormone | 0: Thyroid stimulating hormone 0.27-4.20mUI/L  1: Thyroid stimulating hormone <0.27mUI/L or >4.20mUI/L | 0=1455(90.3)  1=113(7.2)  Missing=44(2.7) |
| Blood Urea | 0: Blood Urea 15-55mOsm/L  1: Blood Urea <15mOsm/L or >55mOsm/L | 0=1495(94.4)  1=89(5.5)  Missing=28(1.7) |
| Vitamin D 25OH | 0: Vitamine D 25OH >30 ng/Ml  0.5: Vitamine D 25OH 10-30 ng/Ml  1: Vitamine D 25OH <10 ng/mL | 0=804(51.5)  0.5=732(46.9)  1=26(1.7)  Missing=50(3.1) |
| Center for Epidemiological Studies-Depression (CES-D) Scale: I was bothered by things that don’t usually bother me | 0: Rarely or None of the Time  0.33: Some or Little of the Time  0.67: Moderately or Much of the Time  1: Most or Almost All the Time | 0=945(62.3)  0.33=305(20.1)  0.67=207(13.7)  1=59(3.9)  Missing=96(6) |
| CES-D Scale: Poor appetite | 0: Rarely or None of the Time  0.33: Some or Little of the Time  0.67: Moderately or Much of the Time  1: Most or Almost All the Time | 0=1184(78.1)  0.33=190(12.5)  0.67=109(7.2)  1=33(2)  Missing=96(6) |
| CES-D Scale: I had trouble keeping my mind on what I was doing | 0: Rarely or None of the Time  0.33: Some or Little of the Time  0.67: Moderately or Much of the Time  1: Most or Almost All the Time | 0=815(53.8)  0.33=396(26.1)  0.67=222(14.6)  1=83(5.5)  Missing=96(6) |
| CES-D Scale: I felt depressed | 0: Rarely or None of the Time  0.33: Some or Little of the Time  0.67: Moderately or Much of the Time  1: Most or Almost All the Time | 0=667(44)  0.33=469(30.9)  0.67=256(16.9)  1=124(8.2)  Missing=96(6) |
| CES-D Scale: I felt that everything I did was an effort | 0: Rarely or None of the Time  0.33: Some or Little of the Time  0.67: Moderately or Much of the Time  1: Most or Almost All the Time | 0=768(50.7)  0.33=447(29.5)  0.67=203(13.4)  1=98(6.5)  Missing=96(6) |
| CES-D Scale: My sleep was restless | 0: Rarely or None of the Time  0.33: Some or Little of the Time  0.67: Moderately or Much of the Time  1: Most or Almost All the Time | 0=489(32.3)  0.33=398(26.3)  0.67=387(25.5)  1=242(16)  Missing=96(6) |
| CES-D Scale: I was happy | 0: Rarely or None of the Time  0.33: Some or Little of the Time  0.67: Moderately or Much of the Time  1: Most or Almost All the Time | 0=557(36.7)  0.33=583(38.5)  0.67=252(16.6)  1=124(8.2)  Missing=96(6) |
| CES-D Scale: I felt lonely | 0: Rarely or None of the Time  0.33: Some or Little of the Time  0.67: Moderately or Much of the Time  1: Most or Almost All the Time | 0=793(52.3)  0.33=341(22.5)  0.67=240(15.8)  1=142(9.4)  Missing=96(6) |
| CES-D Scale: I felt sad | 0: Rarely or None of the Time  0.33: Some or Little of the Time  0.67: Moderately or Much of the Time  1: Most or Almost All the Time | 0=653(43.1)  0.33=511(33.7)  0.67=255(16.8)  1=97(6.4)  Missing=96(6) |
| CES-D Scale: I could not get going | 0: Rarely or None of the Time  0.33: Some or Little of the Time  0.67: Moderately or Much of the Time  1: Most or Almost All the Time | 0=758(50)  0.33=450(29.7)  0.67=225(14.8)  1=83(5.5)  Missing=96(6) |
| IADL - Ability to Use Telephone | 0: Operates telephone on own initiative looks up and dials numbers  0: Dials a few well-known numbers  0: Answers telephone, but does not dial  1: Does not use telephone at all | 0=1464(98.1)  1=29(1.9)  Missing=119(7.4) |
| IADL – Shopping | 0: Takes care of all shopping needs independently  1: Shops independently for small purchases  1: Needs to be accompanied on any shopping trip  1: Completely unable to shop | 0=1440(96.5)  1=53(3.5)  Missing=119(7.4) |
| IADL – Cooking | 0: Plans, prepares, and serves adequate meals independently  1: Prepares adequate meals if supplied with ingredients  1: Heats and serves prepared meals or prepares meals but does not maintain adequate diet  1: Needs to have meals prepared and served | 0=1399(93.7)  1=94(6.3)  Missing=119(7.4) |
| IADL – Housekeeping | 0: Maintains house alone with occasion assistance (heavy work)  0: Performs light daily tasks such as dishwashing, bed making  0: Performs light daily tasks, but cannot maintain acceptable level of cleanliness  0: Needs help with all home maintenance tasks  1: Does not participate in any housekeeping tasks | 0=1297(88.4)  1=171(11.6)  Missing=144(8.9) |
| IADL – Laundry | 0: Does personal laundry completely  0: Launders small items, rinses socks, stockings, etc  1: All laundry must be done by others | 0=1250(83.8)  1=242(16.2)  Missing=120(7.4) |
| IADL – Transportation | 0: Travels independently on public transportation or drives own car  0: Arranges own travel via taxi, but does not otherwise use public transportation  0: Travels on public transportation when assisted or  accompanied by another  1: Travel limited to taxi or automobile with assistance  of another  1: Does not travel at all | 0=1465(98.2)  1=27(1.8)  Missing=120(7.4) |
| IADL– Ability to Handle Finances | 0: Manages financial matters independently (budgets, writes checks, pays rent and bills, goes to bank); collects and keeps track of income  0: Manages day-to-day purchases, but needs help with  banking, major purchases, etc  1: Incapable of handling money | 0=1483(99.3)  1=10(0.7)  Missing=119(7.4) |
| Nutrition: Kcal intake (daily) | 0: 1300-2500 kacl/day  1: <1300 kcal/day or >2500 kacl/day | 0=1109(77.4)  1=323(22.6)  Missing=180(11.2) |
| EQ5D5L – Mobility | 0: autonomous  0.33: mildly dependent  0.67: moderately dependent  1.00: totally dependent | 0=1211(79.3)  0.33=197(12.9)  0.67=98(6.4)  1=22(1.4)  Missing=84(5.2) |
| EQ5D5L – Self care | 0: Excellent  0.33: Good  0.66: Fair  1: Poor | 0=1424(93.2)  0.33=73(4.8)  0.67=22(1.4)  1=9(0.6)  Missing=84(5.2) |
| EQ5D5L – Trouble with usual activity Scale | 0: no trouble  0.33: mild trouble  0.66: moderate trouble  1.00: severe trouble | 0=1155(75.4)  0.33=274(17.9)  0.67=80(5.2)  1=22(1.4)  Missing=81(5) |
| EQ5D5L – Pain | 0: no pain  0.33: mild pain  0.66: moderate pain  1: severe pain | 0=729(47.7)  0.33=506(33.1)  0.67=233(15.2)  1=60(3.9)  Missing=84(5.2) |
| EQ5D5L – Anxiety/depression | 0: no anxiety/depression  0.33: mild anxiety/depression  0.66: moderate anxiety/depression  1: severe/extreme anxiety/depression | 0=588(36.5)  0.33=561(36.6)  0.67=320(20.9)  1=62(4)  Missing=81(5) |
| EQ5D5L – Self-reported health scale | 0: Excellent  0.25: Very good  0.5: Good  0.75: Fair  1: Poor | 0=332(21.7)  0.25=784(51.2)  0.5=312(20.4)  0.75=79(5.2)  1=24(1.6)  Missing=81(5) |
| St. George Respiratory questionnaire – Cough | 0: Not at all/Only with chest infections  0.33: A few days a week  0.67: Several days a week  1: Most days a week | 0=920(61.4)  0.33=265(17.7)  0.67=153(10.2)  1=161(10.7)  Missing=113(7) |
| St. George Respiratory questionnaire – Phlegm | 0: Not at all/Only with chest infections  0.33: A few days a week  0.67: Several days a week  1: Most days a week | 0=1077(71.8)  0.33=217(14.5)  0.67=102(6.8)  1=103(6.9)  Missing=113(7) |
| St. George Respiratory questionnaire - shortness of breath over the last year | 0: Not at all/Only with chest infections  0.33: A few days a week  0.67: Several days a week  1: Most days a week | 0=1083(72.2)  0.33=239(15.9)  0.67=110(7.3)  1=67(4.5)  Missing=113(7) |
| St. George Respiratory questionnaire – Breathless walking stairs | 0: no  1: yes | 0=1234(82.3)  1=265(17.7)  Missing=113(7) |
| Cardiovascular diseases - patient reported clinical diagnosis of myocardial infraction, stroke, chronic heart failure, dyslipidemia, peripheral obstructive artery disease. | 0: absence  1: presence | 0=1514(93.9)  1=98(6.1)  Missing=0(0) |
| Hypertension - patient reported clinical diagnosis, or directly diagnosed by a physician | 0: absence  1: presence | 0=910(56.5)  1=702(43.5)  Missing=0(0) |
| Chronic Kidney disease - estimated glomerular filtration rate below 60 ml/min, via Modification of Diet in Renal Disease study equation | 0: absence  1: presence | 0=1375(85.3)  1=237(14.7)  Missing=0(0) |
| Diabetes Mellitus type II - according to any of the following conditions: fasting glucose ≥126mg/dL, oral glucose tolerance test >200mg/dL or currently on treatment | 0: absence  1: presence | 0=1362(84.5)  1=250(15.5)  Missing=0(0) |
| Cancer - any non-AIDS related cancer, diagnosed with a biopsy | 0: absence  1: presence | 0=1521(94.4)  1=91(5.6)  Missing=0(0) |
| NAFLD - according to risk factors, liver enzymes, FIB-4, altered liver/spleen ratio at CT scan or suggestive US evaluation | 0: absence  1: presence | 0=1194(74.1)  1=418(25.9)  Missing=0(0) |
| Cirrhosis - defined by a FIB-4 higher than 3.25 | 0: absence  1: presence | 0=1434(89)  1=178(11)  Missing=0(0) |
| COPD - chronic obstructive pulmonary disease diagnosed with a reduction in one second - forced expiratory volume or forced vital capacity ratio less than 0.7 at spirometry performance test | 0: absence  1: presence | 0=1542(95.7)  1=70(4.3)  Missing=0(0) |

|  | | |
| --- | --- | --- |
| **Deficit** | **Coding** | **Frequencies**  **N (%)** |
| CDC- Classification | 0=A  0,5=B  1=C | 0=698(46.2)  0.5=451(29.8)  1=362(24)  Missing=101(6.3) |
| CD4 nadir | 0: <350CD4/mmc  1: >350 CD4/mmc | 0=298(19.4)  1=1236(76.7)  Missing=78(4.8) |
| Duration of HIV | 0: <10years  0.33: 10-20 years  0.67: 20-30 years  1: >30 years | 0=215(13.7)  0.33=366(23.4)  0.67=690(44.1)  1=294(18.8)  Missing=47(2.9) |
| Time between diagnosis and ARV initiation | 0: immediately after diagnosis  1: delayed | 0=448(30.7)  1=1011(69.3)  Missing=153(9.5) |
| History of AIDS malignancy requiring chemotherapy or radiotherapy | 0: no  1: yes | 0=1523(94.5)  1= 89(5.5)  Missing=0(0) |
| 3^rd^ line of ARV or more | 0: no  1: yes | 0=371(23.2)  1=1228(76.8)  Missing=13(0.8) |
| CD4+ absolute cell count | 0: >500CD4/mmc  1: <500CD4/mmc | 0=1268(79.3)  1=330(20.7)  Missing=14(0.9) |
| CD4/CD8 | 0= >1  0.5= 0.8-1  1= <0.8 | 0=621(39.2)  0.5=306(19.3)  1=658(41.5)  Missing=27(1.7) |
| HIV-Viral Load (copies/mL) | 0=undetectable  1=detectable | 0=1345(92)  1=117(8)  Missing=150(9.3) |
| Lipodystrophy | 0=absence  1=presence | 0=455(28.2)  1=1157(71.8)  Missing=0(0) |

| **(C)** | | |
| --- | --- | --- |
| **Deficit** | **Coding** | **Frequencies**  **N (%)** |
| Ethnicity | 1= Italian  0=Else | 1=1152(97.2)  0=33(2.8)  Missing=427(26.5) |
| Education | 1=bachelor/master/university  0.75=High school  0.5=Junior High school  0.25=elementary  0=none | 1=293(21.9)  0.75=484(36.1)  0.5=467(34.9)  0.25=89(6.6)  0=7(0.5)  Missing=272(16.9) |
| Physical activity (IPAq) | 0: 3 or more days of vigorous-intensity activity of at least 20 minutes per day OR 5 or more days of moderate-intensity activity and/or walking of at least 30 minutes per day  1: Low physical activity | 1=781(48.7)  0=822(51.3)  Missing=9(0.6) |
| Occupation | 1=Student, free professional  0.67= employee, shop owner, dealer  0.33=farmer, artisan craftsman, worker  0=unemployed, housewife, retired | 1=298(20)  0.67=531(35.6)  0.33=317(21.2)  0=346(23.2)  Missing=120(7.4) |
| Risk | 1=others  0=IDU | 1=1224(75.9)  0=388(24.1)  Missing=0(0) |
| Marital Status | 1=Married/engaged  0=divorced/separated/single | 1=402(33.8)  0=786(66.2)  Missing=424(26.3) |
| Domestic partnership | 1= with partner, sons, parents, friends  0=alone, nursing home | 1=731(62)  0=448(38)  Missing=433(26.9) |
| Income | 1: >70.000 euro/year  0.5: 36.153-70000 euro/year  0: <36.152 euro/year | 1=26(2.3)  0.5=161(14)  0=959(83.7)  Missing=466(28.9) |
| Alcohol | 1:no alcohol  0.5: mild-moderate consumption  0: high consumption | 1=1132(70.8)  0.5=448(28)  0=20(1.3)  Missing=12(0.7) |
| Smoking | 1=no smoke  0.5=mild-moderate smoker  0=hard smoker | 1=1074(67.2)  0.5=245(15.3)  0=280(17.5)  Missing=13(0.8) |
